# Diphtheria at Marlborough College

**Published:** 1890-11-29

**Authors:** Chas. Wm. Rees Stokes

**Affiliations:** Town Clerk's Office, Tenby


### Editor's Letter-Box.

[Our Correspondents are reminded that prolixity is a great bar to publication, and that brevity of style and concieness of statement greatly facilitate early insertion.]

### DIPHTHERIA AT MARLBOROUGH COLLEGE.

Sir,—I have read with much interest the article on Marlborough College, contained in your paper of the 15th instant, as I have a son at that school, and am naturally anxious that the sanitary arrangements there should be as perfect as possible.

I thoroughly agree with every word you have said in the article, and hope you will bring public opinion to bear to compel the authorities to follow out your recommendations, particularly as you will see by the enclosed circular that diphtheria has again broken out there, the authorities easing their consciences by believing that it arose through that unfortunate boy with the nasal complaint.

Dr. Parkes recommended the removal of the present outdoor closets and others being substituted, but so far as I can understand, this has not yet been done, and it is not intended to do anything until the Christmas vacation, whereas Dr. Griffiths, of Swansea, considered that they should be closed, and earth closets or some other kind used in their place.

Hoping you will excuse my troubling you in this matter—but the importance of the subject must be my excuse.—I remain, Sir, your obedient Servant,

Chas. Wm. Rees Stokes.

Town Clerk's Office, Tenby, 22nd November, 1890.

[We can understand the anxiety of Mr. Rees Stokes and other parents on being informed that diphtheria has again broken out at Marlborough. The explanation is given that the boy who is supposed to have originally introduced the diphtheria returned to school a week ago with a medical certificate, and after a careful examination, appeared to be completely cured. A boy in the same dormitory has now developed the complaint in a mild form, and the examination of the other boy shows that his supposed cure was not complete. This is the explanation given by the school authorities. But medical experience interprets the facts very differently from the interpretation here given to them. The following is the obvious statement and explanation of the case from the first: The original boy develops diphtheria at school; he goes home and is cured; the diphtheritic membrane disappears completely; so completely indeed that the family medical attendant sends the boy back with a clean bill of health. When he returns to Marlborough the school doctor examines him, and with critical minuteness we may be sure. The boy, being in the estimation of two critical doctors, thoroughly cured, is passed into his dormitory. Within a week another boy in the same dormitory develops a mild attack of diphtheria; then the original boy is re-examined and found to be not completely cured. In our judgment, the real explanation is that the second boy in the dormitory acquired the disease, not from the original boy, but from some local source of infection still undiscovered, or if not undiscovered, at least unremoved; and that the original boy, although quite cured, and probably free from membrane for fully fourteen days, yet remained with his throat red, a little inflamed, and in a highly susceptible condition. Being exposed in this condition to the action of the poison, he took the disease a second time. His case, we firmly believe, was not one of recrudescence but of fresh infection. In any event the duty of the authorities of Marlborough is now clear. They should call in a sanitary engineer of the highest professional standing. They should instruct him not to think of saving the reputation of the school, but to tell them exactly and fully all the faults and defects which he can find in the school buildings, and also of any new arrangements which he considers would make the sanitation perfect. This is the plain but imperative duty of the school managers at the present crisis. Half measures in cases like the present are not only foolish and unbusiness-like, they are criminal. Every parent who has boys at Marlborough, or indeed at any other school, should combine with every other parent to insist that such preventable and fatal diseases as diphtheria and typhoid fever shall no longer, like dread spectres of death, anywhere haunt the school and the playground where those that are dearest to them are daily exposed to their unseen but mortal attacks.—Ed.]

